# The complete sequence of the mitochondrial DNA and phylogenetic analysis of the marine red alga *Grateloupia elliptica* (Rhodophyta: Halymeniales)

**DOI:** 10.1080/23802359.2022.2160667

**Published:** 2023-02-03

**Authors:** Maheshkumar Prakash Patil, Jong-Oh Kim, Kyunghoi Kim, Young-Ryun Kim

**Affiliations:** a Industry-University Cooperation Foundation, Pukyong National University, Busan, Republic of Korea; b Department of Microbiology, Pukyong National University, Busan, Republic of Korea; c School of Marine and Fisheries Life Science, Pukyong National University, Busan, Republic of Korea; d Department of Ocean Engineering, Pukyong National University, Busan, Republic of Korea; e Marine Eco-Technology Institute, Busan, Republic of Korea

**Keywords:** *cox1* intron Halymeniales, Rhodophyta, mitochondrial genome, phylogenetic analysis, *Grateloupia elliptica*

## Abstract

*Grateloupia elliptica* (Holmes, 1896) is a red alga belonging to the order Halymeniales and phylum Rhodophyta. In this study, the complete mitochondrial DNA (mtDNA) of *G. elliptica* has been described. The complete circular mtDNA of *G. elliptica* was 28,503 bp in length, with an A + T content of 68.78%; it encoded a total of 49 genes, including 20 tRNA, three rRNA, and 26 protein-coding (*CDS*) genes. Phylogenetic analysis based on complete mitochondrial genomes revealed that *G. elliptica* was most closely related to *G. angusta*. The complete mitochondrial sequence of *G. elliptica* will enrich the mitochondrial genome database and provide useful resources for population genetics and evolution analyses.

*Grateloupia elliptica* is a red marine macroalga belonging to the genus *Grateloupia* (Halymeniaceae; Halymeniales; Florideophyceae; Rhodophyta). *G. elliptica* is commonly referred to as an intertidal alga and is found across Korea and Japan (Yang et al. 2013). Recent research has suggested that bioactive substances found in *G. elliptica* may have medicinal benefits (Lee et al. 2021). *Grateloupia* is the largest genus in the Halymeniaceae family, with several species that are difficult to distinguish (Miller et al. 2011). Mitochondrial genes and full mitochondrial DNA (mtDNA) are commonly used for conducting algal species identification, population genetics, and biogeographic investigations (Lee et al. 2009; Kim et al. 2012). The current work examines the complete mtDNA of *G. elliptica* and its evolutionary relationship within the genus.

The *G. elliptica* specimen used in this study was collected from the East Sea, South Korea (37°06′41.5′′N 129°22′45.6′′E) and deposited at the Ecological Restoration Group, Marine Eco-Technology Institute, Busan, South Korea (Young-Ryun Kim, yykim@marine-eco.co.kr) under the voucher number PU-T01-S-MA-02. Total DNA was extracted using DNeasy Blood and tissue kit (Qiagen, Hilden, Germany) following the manufacturer’s instructions. A DNA library was prepared using TruSeq Nano DNA Kit and sequenced on an Illumina platform (HiSeq2500, San Diego, CA); subsequently, 150 bp paired-end reads were generated. The obtained raw data were preprocessed for quality filtering and trimming of low-quality bases and adapters by FastQC. A total of 1,665,114,426 read bases of filtered data were analyzed to generate 11,126,568 reads of sequence; then *De novo* assembly was performed with the Platanus-allee version 2.2.2 (Kajitani et al. 2019) and complete mtDNA annotation was performed using the MFannot tool (https://megasun.bch.umontreal.ca/cgi-bin/mfannot/). A phylogenetic tree was constructed using the whole mitochondrial genome sequences of eight different species. Prior to phylogenetic analysis, the mitochondrial genome was aligned using ClustalW. The evolutionary position of *G. elliptica* was investigated by constructing a phylogenetic tree based on the complete mitochondrial genome using the maximum-likelihood (ML) method and conventional bootstraps analysis (1000 replications) in MEGA11 version 11.0.8 (Tamura et al. 2021).

The complete circular mtDNA (GenBank accession No.: OP479979) of *G. elliptica* was 28,503 bp in length with 68.78% A + T content. The overall base composition was 36.20% for A (10,318 bp), 32.58% for T (9287 bp), 15.95% for G (4546 bp), and 15.27% for C (4352 bp). The mtDNA contained 49 genes, including 20 *tRNA* genes, three *rRNA* genes, and 26 protein-coding (*CDS*) genes. The *CDS* genes consisted of four ATPase subunits, six ribosomal proteins, seven NADH dehydrogenase subunits, three *sdh* subunits, three *cox* subunits, one of each *cob* and *tatC* subunits, and one intronic reading frame (ORF634). In the mtDNA of *G. elliptica* (OP479979), a group II intron is located within the *cox*1 gene, which is similar to *G. angusta* (Kim et al. 2014), *G. taiwanensis* (DePriest et al. 2014), and *G. filicina* (Li et al. 2018). The three *rRNA* genes have lengths of 108 bp (*rrn5* rRNA), 1365 bp (*rns* rRNA), and 2624 bp (*rnl* rRNA). The phylogeny recovered a close relationship between *G. elliptica* with *G. angusta* with maximum bootstrap support (Figure 1). The complete mtDNA sequence of *G. elliptica* may help advance our understanding of the *Grateloupia* evolution.

**Figure 1.**
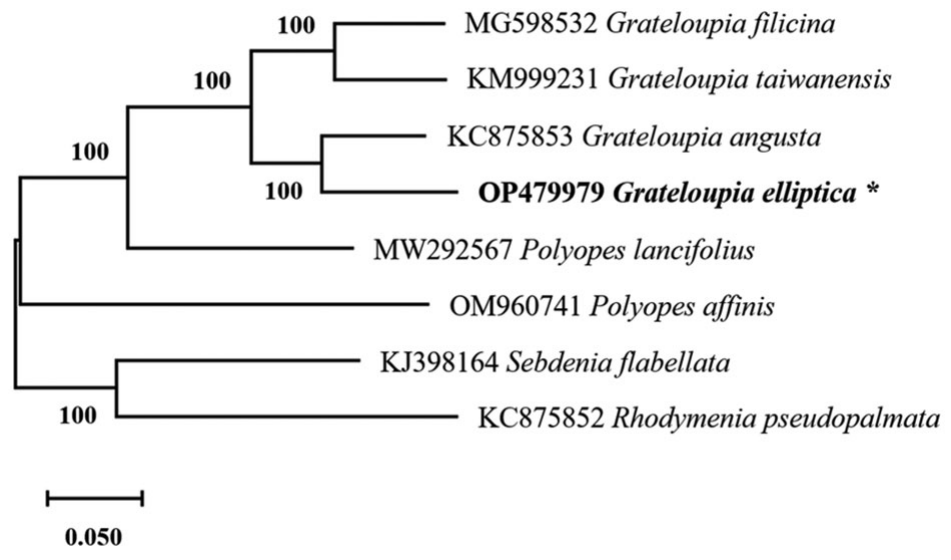
The phylogenetic tree (maximum likelihood) of the all publicly available Halymeniales mitogenome was included and mitogenomes from *Rhodymenia pseudopalmata* (Rhodymeniales) and *Sebdenia flabellata* (Sebdeniales) were included as outgroups. The asterisks beside *Grateloupia elliptica* denote the newly discovered mitochondrial genome.

## Data Availability

The genome sequence data supporting this study’s findings are available in the GenBank database (https://www.ncbi.nlm.nih.gov/) under the accession number OP479979. The associated BioProject, BioSample, and SRA numbers are PRJNA825655, SAMN27532332, and SRR18728769, respectively.
